# Genetic Landscape of Solid Malignant Tumors in a Russian Cohort of Patients

**DOI:** 10.3390/diagnostics16010001

**Published:** 2025-12-19

**Authors:** Iurii K. Slepov, Evgeniy D. Kopylov, Anton A. Turchin, Darya N. Khmelkova, Vladimir S. Kaimonov, Artur A. Isaev, Roman V. Deev

**Affiliations:** 1 Petrovsky National Research Centre of Surgery, 119435 Moscow, Russia; 2 Department of Histology, Petrovsky Medical University, 119435 Moscow, Russia; 3 Center of Genetics and Reproductive Medicine “Genetico”, Public Joint Stock Company, 119571 Moscow, Russia

**Keywords:** comprehensive genomic profiling, cancer, mutations, personalized medicine, Russian population

## Abstract

**Background/Objectives**: Comprehensive genomic profiling (CGP) is a cornerstone of personalized oncology. However, large-scale, systematic data on the somatic mutation spectrum in Russian cancer patients are scarce. This study aimed to characterize the genomic landscape and assess the potential for matched therapy in a Russian cohort of patients with solid tumors. **Methods**: This retrospective study included 204 patients with various solid tumors. CGP was performed using the FoundationOne^®^CDx (FFPE tissue) and FoundationOne^®^Liquid CDx (cfDNA) platforms. The analysis assessed single-nucleotide variants, indels, copy number alterations, gene fusions, tumor mutational burden (TMB), microsatellite instability (MSI), and PD-L1 expression. **Results**: The most frequently mutated genes were *TP53* (61.5%) and *KRAS*. The median TMB was 4.0 mut/Mb and was significantly lower in stage IV tumors. Significant co-occurrence was observed between *KRAS* and *TP53* mutations, as well as between *APC* and *KRAS* mutations, which were particularly characteristic of colorectal cancer. *KRAS* mutations were associated with higher combined positive score (CPS) values in cases with lung cancer. Based on the CGP results, 44% of patients had findings that supported the use of an approved matched targeted therapy or immunotherapy for their tumor type. An additional 36% of patients had alterations indicating potential benefit from off-label targeted therapy. **Conclusions**: This study reveals the distinct genomic characteristics of solid tumors in a Russian cohort and confirms the high clinical utility of CGP for identifying actionable targets. Implementing CGP early in the diagnostic process is a necessary step towards realizing personalized treatment strategies for cancer patients.

## 1. Introduction

Advances in molecular genetic analysis and personalized medicine are bringing about significant changes in the diagnosis and treatment of malignant diseases [1]. Compared to single-gene testing (SGT), comprehensive genomic profiling (CGP) using next-generation sequencing (NGS) provides a more comprehensive molecular portrait by enabling the simultaneous analysis of multiple genes and genomic variants, such as SNVs, copy number alterations, microsatellite instability, and tumor mutational burden [2]. Compared to sequential SGT, CGP offers a more effective and tissue-sparing method; research indicates that it can increase targeted therapy eligibility by 10–15%, improve target marker identification by up to 75%, and find prognostically significant mutations in up to 25% of cases [3,4]. Additionally, in 41–84% of previously tested patients, CGP identifies clinically significant changes overlooked by other techniques, overcoming the significant drawbacks of non-CGP methods, such as incomplete profiling, sequential testing delays, and repeat biopsy risks [5,6,7,8]. Currently available CGP platforms include MSK-IMPACT [9], FoundationOne^®^CDx, FoundationOne^®^Liquid CDx, and laboratory-developed tests [10].

F1CDx has been clinically tested on over 30,000 DNA samples isolated from solid tumor specimens [11]. The molecular genetic test underwent a concurrent review process by the FDA and CMS under the ACCE model, where FDA approval confirmed that the test met criteria for analytical and clinical validity. The CMS assessment, in turn, focused on establishing clinical benefit according to a systematic analysis of treatment outcome measures such as overall survival (OS), progression-free survival (PS), and objective response rate (ORR), based on data from randomized and non-randomized clinical trials [11]. The F1CDx assay tests 324 solid tumor-associated genes. It detects pathogenic variants, copy number alterations (CNAs), and rearrangements. It also reports complex biomarkers like tumor mutational burden (TMB), microsatellite instability (MSI), and, for ovarian cancer, genomic loss of heterozygosity (gLOH).

The success of molecular testing is significantly impacted by factors such as tumor type and the quality of tissue sample preparation. As shown by Lin et al. (2025), in a pan-tumor context, the suitability of samples for analysis using the F1CDx test remains consistently high, with a median value of 92.3% (range: 88.2–96.9% depending on the type of material) [12]. This method is particularly relevant given that routine clinical samples are often characterized by low tumor purity and high genetic heterogeneity due to previous treatment. These factors naturally lead to a decrease in the frequency of allelic variants (VAF), which complicates the detection of significant mutations. An important advantage of F1CDx under these conditions is its high sensitivity. It can identify not only primary driver mutations but also secondary genetic changes linked to therapy resistance [13].

The effectiveness of molecular genetic testing in real-world practice is supported by data in the literature. In a study by Hung et al. (2025), based on the results of CGP, 43 patients (9.4%) diagnosed with lung adenocarcinoma received selected targeted therapy [14]. The median overall survival of patients who received and did not receive selected therapy was 26.1 months and 10.6 months, respectively, indicating the significant role of CGP in identifying genomic alterations and developing effective individualized treatment strategies. F1CDx allows for the replacement of several highly specialized tests with a single one, saving time, resources, and, critically, tumor material. It also helps avoid “contamination” of the report with clinically insignificant findings, which can occur with whole exome or genomic sequencing [11].

This study is timely and significant for personalized oncology, motivated by several critical factors related to CGP’s high efficiency in therapy selection. The genomic landscape of malignant tumors can vary significantly across ethnic and population groups, influenced by genetic characteristics and environmental factors. Currently, there is a significant shortage of large-scale and systematic data characterizing the spectrum of somatic mutations in cancer patients in the Russian Federation. This has created a blind spot for both scientific research and clinical practice. A key objective of personalized oncology is to determine the actionability of genomic alterations. This means defining what proportion of patients with specific genetic aberrations are suitable for existing targeted drugs. This goal is central amid rapid advances in sequencing technology and growing data on genetic predisposition. Obtaining this information will enable comparative analysis with existing databases, such as TCGA and GENIE, to identify unique characteristics of the Russian population, which may be important for the development and registration of new drugs.

## 2. Materials and Methods

### 2.1. Patient Cohort and Sample Collection

This retrospective study included patients who presented to the Genetico Medical Center between January 2023 and October 2025 for comprehensive genomic profiling (CGP) using the FoundationOne CDx and FoundationOne Liquid CDx platforms. Indications for CGP were: advanced solid tumors, disease progression after standard therapy, rare histological types, and evaluation for potential targeted therapy or immunotherapy. DNA samples were obtained from paraffin-embedded DNA (FFPE) or as circulating free DNA (cfDNA) from blood. Clinical and morphological data (age, gender, histological type, date of collection, and analysis) were extracted from the records provided to the medical center.

This retrospective study included patients who presented to the Genetico Medical Center between January 2023 and October 2025 for comprehensive genomic profiling (CGP). Indications for CGP were: advanced solid tumors, disease progression after standard therapy, rare histological types, and evaluation for potential targeted therapy or immunotherapy. The primary inclusion criterion was the availability of a formal report from the Foundation Medicine laboratory.

### 2.2. Sample Submission and Genomic Analysis

The samples were sent to the Roche Foundation Medicine laboratory (Germany) for genomic profiling using the FoundationOne CDx (for FFPE) and FoundationOne Liquid CDx (for cfDNA) platforms. Briefly, the analysis is based on hybrid capture and next-generation sequencing (NGS) of the coding regions of 324 oncogenic genes, identifying single-nucleotide variants, indel mutations, copy number changes, and fusions. Sequencing depth exceeds 500× for tissue and 5000× for plasma-derived cfDNA [11,15]. All samples (FFPE tissue blocks or blood draws for cfDNA) were collected per standard clinical protocols. For tissue samples, before sending, the surface area of the tumor (min. 25 mm^2^) and the percentage of tumor nuclei were assessed (min. 20%). Samples were sent to the Roche Foundation Medicine laboratory (Germany) with associated clinical information. The laboratory’s pre-analytical quality control process was applied to all specimens. For FFPE samples, this includes an re-evaluation of tumor content and assessment of DNA quality. Samples failing this QC (e.g., due to insufficient tumor nuclei, low DNA yield, or excessive fragmentation) are deemed inadequate, and a report is issued stating the sample did not meet the requirements for successful analysis. Only samples that passed this internal QC and generated a full genomic profile report were included in this study. Therefore, the results presented here are based on samples deemed analytically adequate by the testing platform’s validated standards. Genomic profiling was then performed using the FoundationOne CDx (for FFPE) or FoundationOne Liquid CDx (for cfDNA) platforms, as previously described [11,15].

### 2.3. Assessment of Tumor Molecular Characteristics

The following biomarkers were assessed in the study: total mutation burden (TMB), microsatellite instability status (MSI), and PD-L1 expression. TMB was calculated by counting all variants with an allele frequency of 5% or higher (after filtering) and converting the mutation rate per megabase (mut/Mb). MSI was determined using the FB-MSI algorithm of the FoundationOne CDx platform. Briefly, the method involves genomic analysis of over 2000 microsatellite loci and calculating the proportion of unstable loci with somatic alleles that differ from the reference sequence. The ratio of the number of unstable loci to the total number of loci being evaluated is then calculated. Based on the final value, the sample was classified as MSI-High (FB-MSI scores ≥ 0.0124) or microsatellite stable (MSS, FB-MSI scores ≤ 0.0041). Intermediate values were described as “Cannot be Determined” [15].

PD-L1 expression was assessed using immunohistochemical (IHC) staining. Depending on the tumor type and the intended therapy, validated Dako (22C3, 28-8) or Ventana (SP142, SP263) test systems were used to calculate the following possible indicators: tumor proportion score (TPS), tumor cell (TC) score (%), tumor-infiltrating immune cell (IC) score (%), and combined positive score (CPS). Paraffin blocks were used for the analysis. The test was selected taking into account recommendations for a specific diagnosis and a list of potential immunotherapeutic regimens [16].

### 2.4. Interpretation of Genetic Variants and Assessment of Clinical Significance

Predictive markers were defined as molecular alterations with therapeutic significance according to indications documented in FDA, EMA, Swissmedic product labels, or NCCN guidelines, as well as those related to targets with level I/II evidence according to the ESMO ESCAT clinical significance scale. Therapy recommendations and resistance markers were derived from FoundationOne summary reports. The oncoKB database was used to annotate the effect of mutations [17].

### 2.5. Statistical Analysis

Descriptive statistics, frequency distributions, and biomarker distributions were determined using R v4.5.1 software. Comparisons between subgroups were performed using the χ^2^ test for categorical variables and the Brunner–Munzel test for nonparametric data. Spearman’s correlation was used to assess associations between two discrete variables. Statistical significance was set at *p* < 0.05.

## 3. Results

### 3.1. Characteristics of the Patient Cohort

**Figure 1:**
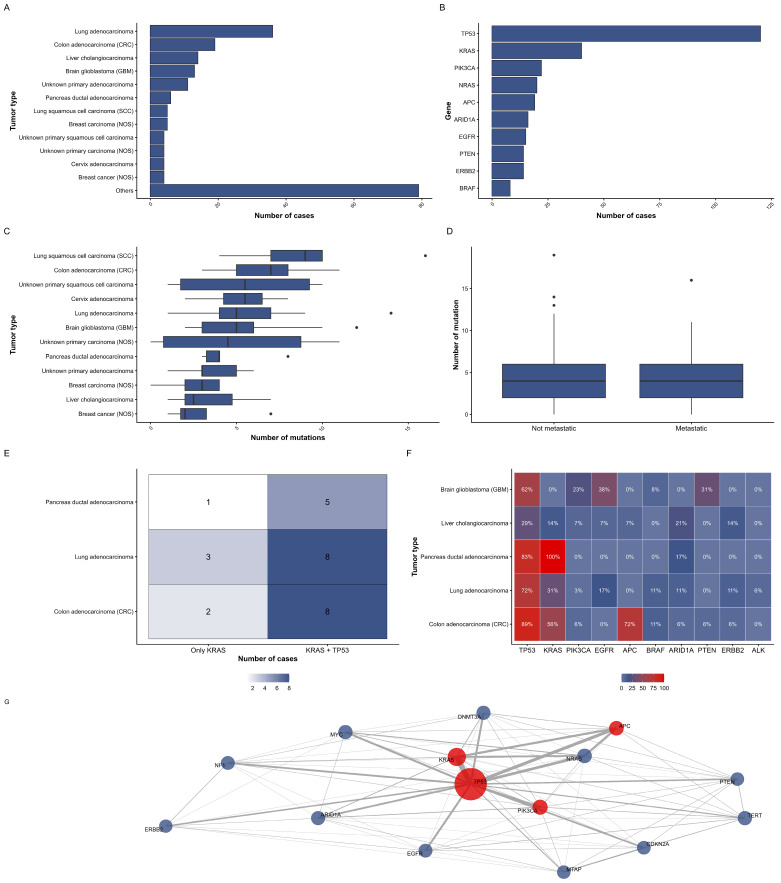
(**A**) Distribution of included cases according to 12 most represented nosologies; (**B**) number of cases with mutations in 10 most frequent genes; (**C**) number of mutations in 12 most represented nosologies; (**D**) comparison of cases with and without identified metastases based on the number of mutations; (**E**) co-occurrence table for *KRAS* and *TP53*; (**F**) mutation frequency of 10 most common genes in 5 most represented localizations; (**G**) network analysis of co-occurrence of 15 most frequent mutations. The node size is directly proportional to the mutation frequency, while the edge thickness is inversely proportional to the co-occurrence frequency.

This study comprised 204 patients with solid malignant neoplasms. For 161 patients (78.9%), clinical data were available, including the date of diagnosis, TNM stage of the disease, information on surgical treatment, lines of systemic therapy, and responses to it. Among the 161 patients with clinical data, information on systemic therapy was available for 89 patients (43.6%) (Table 1). The sample included 66 different histological types of tumors. The most prevalent nosologies were lung adenocarcinoma, colon adenocarcinoma, cholangiocarcinoma, glioblastoma, adenocarcinoma of unknown primary location, ductal carcinoma of the pancreas, breast carcinoma, and lung squamous cell carcinoma (for the complete list, see Supplementary Table S1). The remaining 58 histological variants were represented by 1 to 4 cases each, reflecting the high nosological heterogeneity of the cohort (Figure 1A).

### 3.2. Genomic Landscape

#### 3.2.1. Overall Mutation Burden and Distribution

Comprehensive genomic profiling revealed mutations in the p53 tumor suppressor gene most frequently detected. The subsequent most prevalent mutations were observed in the KRAS gene. The 10 most frequently detected genes are listed in Figure 1B, with full details provided in Supplementary Table S2.

The median number of mutations observed was four per patient. The highest number of mutations detected was 19, identified in a 35-year-old female patient with anaplastic astrocytoma. The distribution of mutation counts across the main tumor types is shown in Figure 1C, and the underlying data per tumor type are available in Supplementary Table S3. No statistically significant differences in the number of mutations were observed between patients with and without metastases (Brunner–Munzel Test Statistic = −0.83809, *p*-value = 0.4034, Figure 1D). The investigation revealed no statistically significant associations between age and the number of mutations, nor between the number of chemotherapy lines and the number of mutations.

#### 3.2.2. Co-Occurrence of Key Driver Mutations

*KRAS* mutations were found to be associated with *TP53* mutations (χ^2^ = 7.3, *p*-value = 0.04). Alterations in both genes were detected in 72.5% of cases with *KRAS* mutations and in 24.2% of cases with *TP53* mutations (Figure 1E). The most prevalent nosologies in which defects in both genes were identified included colon adenocarcinoma, lung adenocarcinoma, and pancreatic ductal adenocarcinoma (see Figure 1F).

The results of the association analysis between *TP53*, *KRAS*, and *APC* mutations in a pooled cohort (*n* = 204) have revealed the most pronounced and significant association to be the co-occurrence of *APC* and *KRAS* mutations (χ^2^ = 34.7, *p* < 0.0001). This co-occurrence was detected in 63% of cases with an *APC* mutation and 30% of cases with a *KRAS* mutation. Furthermore, a significant association was identified between *APC* and *TP53* (χ^2^ = 6.8, *p* = 0.004), which exhibited a marked asymmetry in co-occurrence: for instance, 90% of tumors with an *APC* mutation also have a *TP53* mutation, while only 14% of *TP53*-mutated tumors have a mutation in *APC*. Analysis with localization revealed that frequent co-mutations of *APC*, *KRAS*, and *TP53* are characteristic of colorectal cancer (CRC), where a triple mutation occurs in 42% of patients, whereas in other nosologies (lung adenocarcinoma, cholangiocarcinoma, glioblastoma), the frequency and co-occurrence of these mutations are significantly lower and statistically insignificant.

#### 3.2.3. Functional Classification of Genomic Alterations

**Figure 2:**
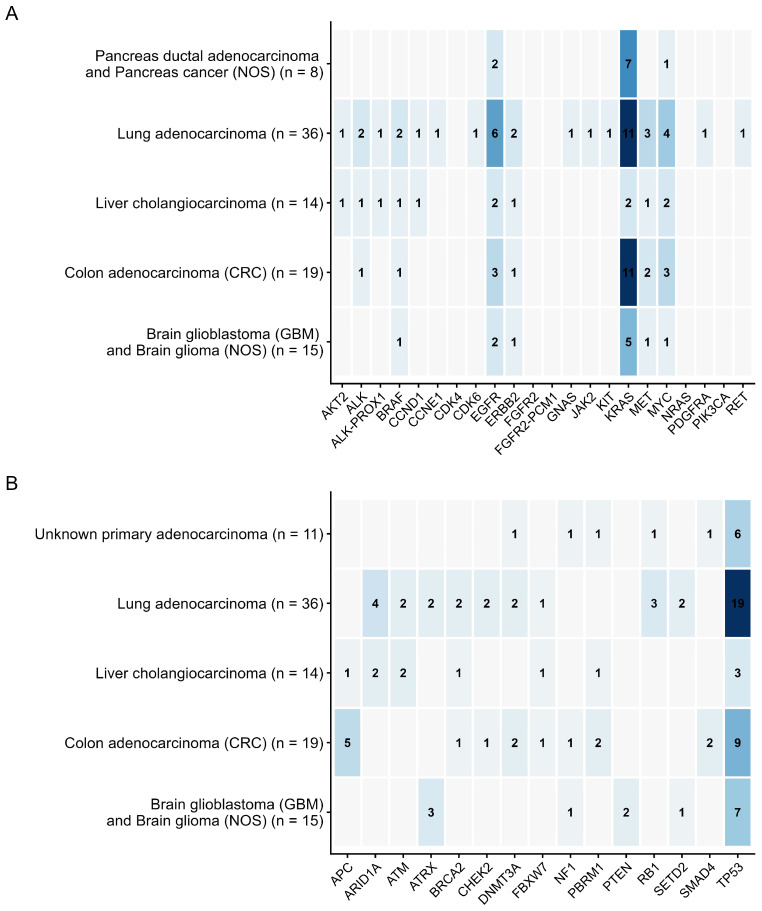
Distribution of mutations for five nosologies with the highest number of mutations of each type. (**A**) Activating mutations. (**B**) Loss-of-function mutations and likely loss-of-function mutations.

Among the 928 genetic variants identified, 162 (17.5%) were activating mutations, including missense mutations in oncogenes (e.g., *KRAS* G12D, *BRAF* V600E), amplifications (e.g., *ERBB2* amplification, *MYC* amplification), fusion genes (e.g., *EML4-ALK*, *ROS1-GOPC*), and activating deletions and insertions (e.g., *EGFR* exon 19 deletion). Inactivating variants, including nonsense (*TP53* R175*), frameshift (*TP53* L265fs*4), and splice site mutations, gene or exon deletions (*PTEN* loss, *CDKN2A* loss), and mutations in tumor suppressor genes (*APC*, *RB1*, *NF1*), accounted for 766 (82.5%) of the total (Figure 2).

### 3.3. Immune Profile

**Figure 3:**
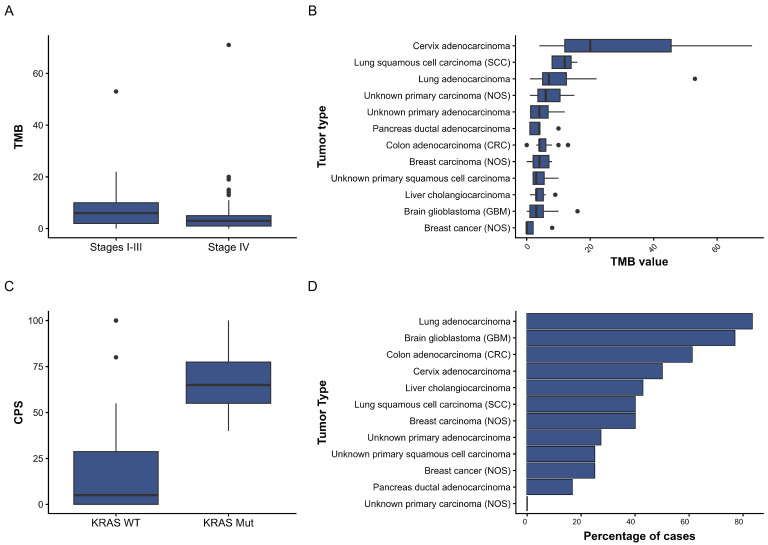
(**A**) Comparison of TMB values between tumors of differing stages; (**B**) distribution of TMB values for the 12 most prevalent localizations in the sample; (**C**) comparison of CPS values between tumors with and without mutations in the *KRAS* gene; (**D**) frequency of therapy selection based on the results of comprehensive genomic profiling for the 12 most prevalent nosologies in the sample.

A comprehensive genomic analysis yielded values for key immune biomarkers and tumor characteristics. The median total mutational burden (TMB) was 4.0 mutations per megabase, with an average value of 5.7 mutations/Mb, and a maximum recorded value of 71 (female, age 44 years, cervical adenocarcinoma). The highest values were observed in cases of cervical adenocarcinoma, squamous cell carcinoma, and lung adenocarcinoma (Figure 3B). Stage IV tumors exhibited a substantially diminished mutational burden in comparison to stage I–III tumors (Brunner–Munzel Test Statistic = −2.7886, *p*-value = 0.006179) (Figure 3A). This trend was observed in the five most represented localisations, with the exception of liver cholangiocarcinoma (Figure S1). Considering that 12 of 32 cases with TMB greater than and equal to 10 were lung adenocarcinoma, this localization is the most representative for our sample.

Microsatellite status distribution was dominated by microsatellite-stable tumors (MS-Stable—147 cases), with a high MSI level detected in only one patient (a 15-year-old girl with glioblastoma). The intermediate category “MSI-High Not Detected” occurred in 33 tumors, and the status was not determined for 23 samples.

The expression of tumor immune biomarkers was measured using CPS. This measurement was taken from 32 patients, with a median CPSs of 12.0 and a maximum CPS of 100.0. Patients with a KRAS mutation demonstrated higher CPS scores compared to those with wild-type KRAS (median CPS 65 vs. 5, Brunner–Munzel Test Statistic = 4.7239, *p* < 0.0001) (Figure 3C). Data on CPS in the presence of KRAS mutation were available only for lung cancer cases (*n* = 4).

TPS was evaluated in 115 patients, with a median TPS of 0.0, a mean value of 12.4, and a range from 0 to 95. The majority of samples exhibited low PD-L1 expression. Tumor-infiltrating immune cells (IC) were observed in a mere eight patients, exhibiting a median of 0.0 and a mean of 0.5 (ranging from 0 to a maximum of 2.0). The TC was determined in 45 patients; the median was 1.0, the mean was 17.7, and the maximum recorded value was 100.0. No differences were identified for any of the parameters when the sample was divided based on the presence of metastases.

### 3.4. Treatment Recommendations

Of the 204 patients included in this study, 90 (44%) met the eligibility criteria for therapy approved for the tumors in the specified location. The most prevalent tumor types selected for therapy were lung adenocarcinoma, glioblastoma, and colon adenocarcinoma (Figure 3D). Of the 90 patients, immunotherapy alone was recommended in 17 cases, with a TMB > 10 Muts/Mb serving as the indication. In five cases, the TMB value was below 10 Muts/Mb. However, the IHC results with anti-PD-L1 antibodies indicated the possibility of immunotherapy.

In 74 cases (36%), therapy that had been approved for tumors in other locations was deemed eligible, based on the identified molecular profile. For 13 patients, this was the sole viable option, as targeted agents recommended for their tumor type were unavailable. Most of these patients had malignancies that were underrepresented in the sample, including anal canal cancer and prostate cancer. However, molecularly compatible candidates were identified for colon adenocarcinoma (1 case out of 19, 5%), cholangiocarcinoma (2 cases out of 14, 14%), and head and neck tumors (2 cases out of 5, 40%), in which no target mutations for approved drugs were identified.

### 3.5. Timing of CGP Relative to Treatment Lines in Patients with Actionable Findings

To assess the real-world integration of CGP results into the therapeutic pathway, the subset of patients for whom CGP supported an approved targeted therapy (*n* = 90) was analyzed. The median time from diagnosis to CGP for this group was 1.07 years (IQR: 0.53–2.86). Critically, only 28 patients (31%) received CGP in time to inform first-line therapy. For the remaining 62 patients (69%), the actionable genomic finding was identified only after they had already received at least one line of non-targeted systemic therapy (median = 1; maximum = 3).

## 4. Discussion

This study presents the results of a genetic profile of solid tumors identified in the Russian population. Notably, the average age of patients in our cohort was approximately 10 years younger than in similar published studies using identical methodology, where average ages were reported as 64 and 67 years [18,19]. The similar tumor spectrum suggests two possible factors: earlier tumor development in this cohort or earlier detection of the disease. Our study provides concrete, cohort-derived data underscoring the need for earlier CGP implementation. While we demonstrated a high overall rate of actionable findings (44%), the timing analysis reveals a critical implementation gap. Among patients with an actionable target, nearly 70% had already undergone at least one line of conventional therapy before CGP was performed. This delay means that for most patients in our real-world cohort, the potential benefit of precision medicine, to guide the most effective initial therapy, was missed. This pattern aligns with the observation that 30% of our total cohort underwent CGP only after 2–5 lines of chemotherapy, suggesting that genomic profiling is often utilized as a late-line diagnostic rather than a foundational tool. Our data quantitatively show that actionable targets are present at high frequency but are frequently discovered too late to optimize first-line treatment selection. In the pursuit of effective diagnostic strategies, it is imperative to prioritize research methods that significantly enhance patient outcomes. Specifically, these methods should focus on improving the quality of life for patients while also addressing the associated economic burdens.

The nosological structure of the tumors was found to be consistent with that observed in other studies that have previously been published, in which the genetic profile was assessed in geographically and ethnically mixed cohorts. A review of the literature revealed that this method was most frequently used in cases of non-small-cell lung cancer, colorectal cancer, breast cancer, ovarian cancer, prostate cancer, and others [18,19]. This configuration is principally driven by the necessity for tumor genetic profiling to inform targeted therapeutic interventions, a principle that is firmly established in international guidelines [20].

The mutational profile of the tumors studied differed from that observed in the Japanese population [18]. In the context of the present cohort, no targetable mutations in the ERBB2 gene were identified in the five cases of gastric cancer (three of which exhibited metastases). Furthermore, among the 19 cases of colon adenocarcinoma (12 cases with identified metastases) examined, only 1 (5.3%) exhibited an alteration in the ERBB2 gene that was associated with the initiation of therapy.

The median mutational load in the present study was determined to be 4.0 mutations per megabase. For non-small-cell lung cancer, the figure was 10.0 mutations/Mb, and for colorectal cancer, 4.0 mutations/Mb. Consequently, the majority of cases included in this study are classified as low mutational load [19,21], thereby underscoring the significance of mutation detection for the purpose of targeted therapy.

Additionally, it has been demonstrated that certain mutations can enhance the efficacy of tumor response to immunotherapy. The present study established a correlation between the presence of a KRAS mutation and elevated values on one of the tests utilized for the purpose of predicting response to immunotherapy, CPS. This observation is consistent with the literature data demonstrating a superior tumor response to immunotherapy in the presence of a KRAS mutation [22,23,24]. This correlation is noteworthy as it reinforces emerging evidence that oncogenic drivers like KRAS can shape the immune contexture of tumors. Our data corroborate findings from larger genomic landscapes, including the study by Salem et al., which described complex interrelations between KRAS mutations and various immuno-oncology biomarkers. This consistency across studies strengthens the hypothesis that KRAS-mutant cancers may represent a distinct subset with specific implications for immunotherapy strategies [25]. However, the present study included only 4 observations with both CPS values and KRAS mutations, all from lung cancer cases. The total number of cases with a known CPS value was 32. This limited number of observations with CPS data restricts the strength and generalizability of the conclusions, especially across other tumor types. Larger studies with more comprehensive data are needed to confirm these findings.

It should be noted that in the analyzed cohort, alternative variants were most frequently detected (61.5% of cases) in the tumor suppressor gene TP53, a fact that has not received significant attention previously [18]. It has been demonstrated that such mutations are predominantly associated with mutations in genes that regulate the cell cycle, including KRAS (14%), NRAS (2%), and another tumor suppressor, APC (8%), among others. Furthermore, in specific locations of solid tumors, triple mutations are observed in nearly 40% of cases (CRC). These data are consistent with publications showing that co-mutation of KRAS and TP53 is characteristic of non-small-cell lung cancer [26], and it can be considered a prognostic marker for the effectiveness of immune checkpoint blockade. A fundamental research question involves identifying indications for targeted therapy to ascertain the potential benefits for patients. In the present study, 44% of patients exhibited a positive response to the test; for CRC and liver cholangiocarcinoma, this figure was 11 and 6 out of 204 cases (5% and 3%, respectively), thus rendering the obtained data comparable to other results [19]. In 2023, 121,425 cases of stage IV cancer were diagnosed in Russia. Consequently, targeted therapy could be a viable option for 53,427 patients [27]. Given the predicted 19.5% increase in incidence by 2036, the implementation of genomic profiling in public healthcare institutions is becoming a key issue in modern healthcare [28].

This study provides the first systematic description of the mutation spectrum in a Russian cohort. It confirms a high frequency of actionable therapeutic targets and reveals several intriguing associations, such as between TMB and disease stage. These findings, which may reflect driver mutation-driven tumor progression, warrant priority investigation in larger, independent cohorts.

It is important to emphasize that the primary objective of this study was descriptive and hypothesis-generating rather than confirmatory in nature. Descriptive cohort studies employing comprehensive genomic profiling play a critical role in mapping the mutational landscape of a population, particularly in the context of limited systematic data available for the Russian population. The identification of mutation frequencies, co-mutation patterns, and potentially actionable alterations in real-world clinical practice provides a valuable foundation for hypothesis generation, which can subsequently be tested in larger prospective studies.

The data presented indicate that the genetic profile and actionable gene variants vary across different populations. It is therefore imperative that comprehensive genetic testing be implemented in routine clinical practice at the early stages of diagnosis if a personalized approach to cancer patient treatment is to be adopted, which is likely to improve survival.

## Figures and Tables

**Table 1:** Characteristics of patients in the study population.

Characteristic	Value
Gender: *n* (%)	
Male	91 (44.6)
Female	113 (55.4)
Age at the time of testing (mean ± sd)	52.11 ± 16.5
Number of years since diagnosis: median (Q1–Q3)	1.07 (0.53–2.86)
Stage: *n* (%)	
I	11 (5.4)
II	14 (6.9)
III	44 (21.6)
IV	92 (45.1)
Data are not available	43 (21.1)
Presence of metastases: *n* (%)	
No	129 (63.2)
Yes	75 (36.8)
Material used for DNA extraction: *n* (%)	
FFPE	170 (83.3)
Blood	34 (16.7)
Number of chemotherapy lines: *n* (%)	
1	40 (19.6)
2	18 (8.8)
3	27 (13.2)
4	2 (0.9)
5	2 (0.9)

## Data Availability

The data presented in this study are available on request from the corresponding author. The data are not publicly available due to privacy.

## Supplementary Materials

The following supporting information can be downloaded at: https://www.mdpi.com/article/10.3390/diagnostics16010001/s1, Table S1: number of cases of different localizations; Table S2: number of cases with mutation in the assessed genes; Table S3: distribution of altered genes by localization. Figure S1: Tumor mutational burden (TMB) in stages I–III versus stage IV across different tumor types.

## Abbreviations

The following abbreviations are used in this manuscript:

CGP	Comprehensive Genomic Profiling
FFPE	Formalin-Fixed, Paraffin-Embedded
cfDNA	Cell-free DNA
TMB	Tumor Mutational Burden
MSI	Microsatellite Instability
CPS	Combined Positive Score
NGS	Next-Generation Sequencing
SGT	Single-Gene Testing
SNV	Single Nucleotide Variant
F1CDx	FoundationOne® CDx
FDA	Food and Drug Administration
EMA	European Medicines Agency
CMS	Centers for Medicare & Medicaid Services
ACCE	Analytic validity, clinical validity, clinical utility, and associated ethical, legal, and social implications
OS	Overall Survival
PS	Progression-free Survival
ORR	Objective Response Rate
CNA	Copy Number Alterations
gLOH	genomic Loss of Heterozygosity
VAF	Frequency of Allelic Variants
IHC	immunohistochemical
TPS	Tumor Proportion Score
TC	Tumor Cell
IC	Tumor-Infiltrating Immune Cell
NCCN	National Comprehensive Cancer Network
ESMO	European Society for Medical Oncology
ESCAT	ESMO Scale for Clinical Actionability of molecular Targets
CRC	Colorectal Cancer
QC	Quality Control
