# Supplementary material for: Genetic Landscape of Solid Malignant Tumors in a Russian Cohort of Patients
Source: Diagnostics (Basel). 2025 Dec 19;16(1):1. doi: 10.3390/diagnostics16010001 (PMC12785937; doi:10.3390/diagnostics16010001)
Supplement: Supplementary file 1 [file diagnostics-16-00001-s001.zip › Figure S1.pdf]

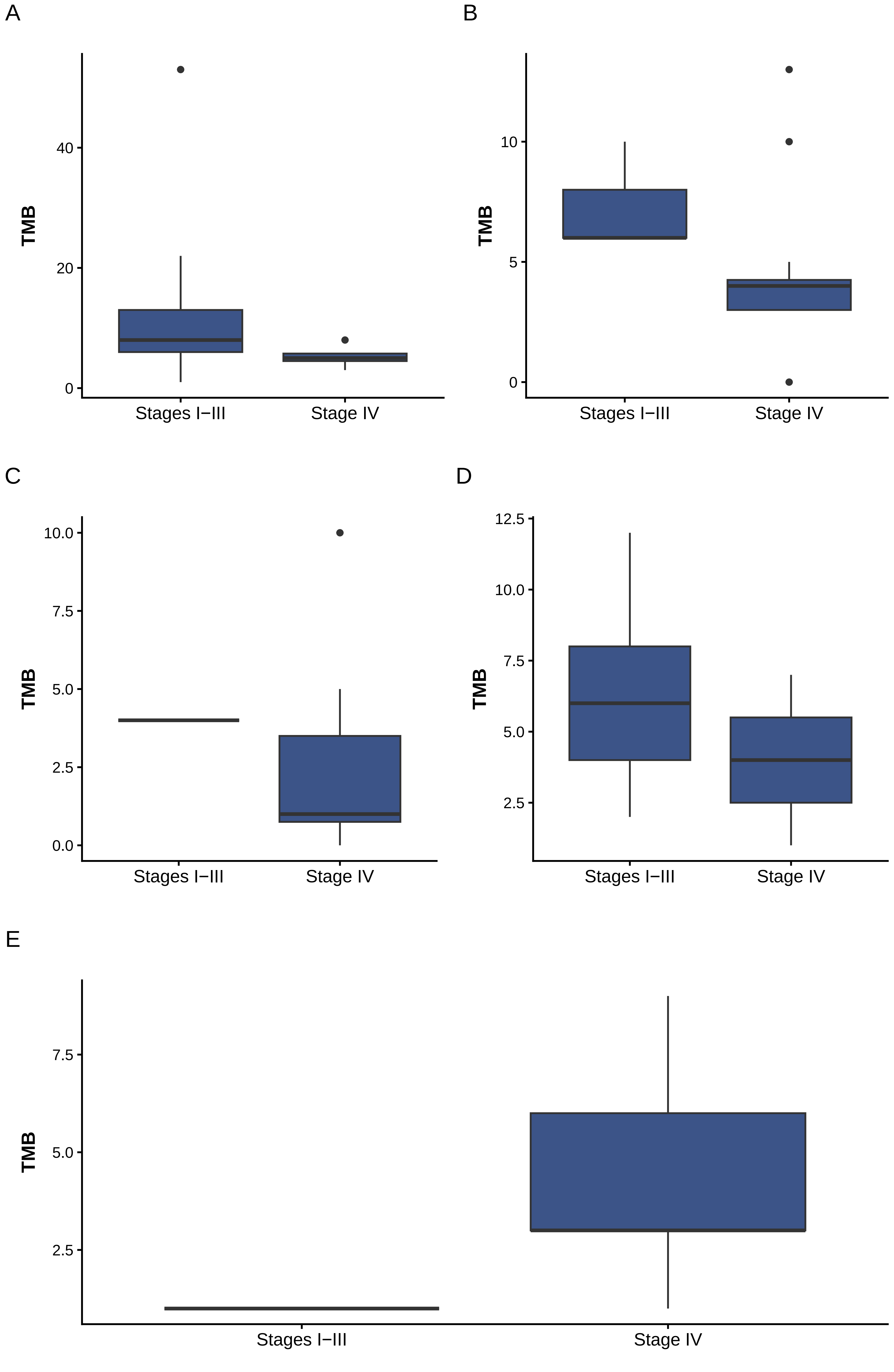

Figure S1. Tumor mutational burden (TMB) in stages I–III versus stage IV across different tumor types. A: Lung adenocarcinoma; B: Colon adenocarcinoma (CRC); C: Brain glioblastoma (GBM); D: Unknown primary adenocarcinoma; E: Liver cholangiocarcinoma
